# HIV-1 Replication through hHR23A-Mediated Interaction of Vpr with 26S Proteasome

**DOI:** 10.1371/journal.pone.0011371

**Published:** 2010-06-29

**Authors:** Ge Li, Robert T. Elder, Larisa Dubrovsky, Dong Liang, Tatiana Pushkarsky, Karen Chiu, Tao Fan, Josephine Sire, Michael Bukrinsky, Richard Y. Zhao

**Affiliations:** 1 Department of Pathology, Department of Microbiology-Immunology, Institute of Human Virology, University of Maryland School of Medicine, Baltimore, Maryland, United States of America; 2 Children's Memorial Research Center, Northwestern University Feinberg School of Medicine, Chicago, Illinois, United States of America; 3 Department of Microbiology and Tropic Medicine, George Washington University, Washington, D. C., United States of America; 4 Pathogénie des Infections à Lentivirus, INSERM U372, Marseille, France; University of Minnesota, United States of America

## Abstract

HIV-1 Vpr is a virion-associated protein. Its activities link to viral pathogenesis and disease progression of HIV-infected patients. *In vitro*, Vpr moderately activates HIV-1 replication in proliferating T cells, but it is required for efficient viral infection and replication *in vivo* in non-dividing cells such as macrophages. How exactly Vpr contributes to viral replication remains elusive. We show here that Vpr stimulates HIV-1 replication at least in part through its interaction with hHR23A, a protein that binds to 19S subunit of the 26S proteasome and shuttles ubiquitinated proteins to the proteasome for degradation. The Vpr-proteasome interaction was initially discovered in fission yeast, where Vpr was shown to associate with Mts4 and Mts2, two 19S-associated proteins. The interaction of Vpr with the 19S subunit of the proteasome was further confirmed in mammalian cells where Vpr associates with the mammalian orthologues of fission yeast Mts4 and S5a. Consistently, depletion of hHR23A interrupts interaction of Vpr with proteasome in mammalian cells. Furthermore, Vpr promotes hHR23A-mediated protein-ubiquitination, and down-regulation of hHR23A using RNAi significantly reduced viral replication in non-proliferating MAGI-CCR5 cells and primary macrophages. These findings suggest that Vpr-proteasome interaction might counteract certain host restriction factor(s) to stimulate viral replication in non-dividing cells.

## Introduction

HIV-1 viral protein R (Vpr) is a virion-associated protein with an average length of 96 amino acids (∼15 kD). Vpr displays several distinct activities in host cells, including cytoplasmic-nuclear shuttling [1], induction of cell cycle G2 arrest [2] and cell killing [3]. The cell cycle G2 arrest induced by Vpr is thought to suppress human immune functions by preventing T cell clonal expansion [4] and to provide an optimized cellular environment for maximal levels of viral replication [5]. Vpr-induced G2 arrest also leads to apoptosis. It is unclear at present what is the biological significance of this effect but it may contribute to the depletion of CD4+ T cells in HIV-infected patients [6]. The cytoplasmic-nuclear shuttling is believed to contribute to nuclear transport of the viral pre-integration complex (PIC)[1], [7].

HIV-1 Vpr contributes to viral replication at least in two different ways. First, in proliferating cells, Vpr promotes viral replication by blocking cell proliferation of HIV-infected T-cells and arresting them in G2 phase of the cell cycle, where the viral replication reaches maximal levels [5]. Contribution of Vpr to viral replication in proliferating T-cells, however, is relatively small *in vitro* as depletion of *vpr* gene from the viral genome typically results in a 2–4 fold reduction of viral replication [5]. On the other hand, Vpr is essential for efficient viral replication in non-dividing cells such as macrophages [8]. Why the requirement for Vpr differs in these two cell types is not well understood.

Noticeably, a recent paper showed that the differential requirement for Vpr is not due to the cell proliferation status, as infection of arrested T-cells by Vpr(−) HIV-1 reduced viral replication by 2-fold compared to Vpr(+) virus [9], which is essentially the same level of reduction observed in proliferating cells. In addition, Vpr participates in nuclear import of PIC in T cells in a similar manner as it does in macrophages, and nuclear import through the nuclear pore is essential for HIV replication in both cell types [10].

Recently, several reports demonstrated that the activity of Vpx, an SIV protein similar to Vpr, stimulates reverse transcription by counteracting a yet unidentified cellular restriction factor [11], [12]. Interestingly, expression of Vpx stimulates replication in macrophages not only of lentiviruses, including HIV-1, but also gamma retroviruses such as MLV [13]. The finding that Vpx stimulates replication in macrophages of Vpr-expressing HIV-1 [11], [12] suggests that either Vpr is a weak inhibitor of a Vpx-targeted restriction factor, or that Vpr may target other host restriction factors that are different from those targeted by Vpx. The ability of Vpx to counteract the restriction of HIV-1 and SIV infection in macrophages depends on DDB1, a subunit of the VprBP-associated E3 ligase [11], [12]. A DDB1-Vpr fusion could partially substitute for the role of Vpx [11]. These findings suggest that Vpr may work in concert with an ubiquitin-proteasome system to limit cellular restriction factor(s) that is normally resistant to HIV infection in macrophages.

The proteasome (or 26S proteasome) is a large multi-subunit protein complex, which is made up of two distinct subcomplexes, the 20S catalytic core and the 19S regulatory cap [14]. The proteasome is responsible for ubiquitin (Ub)-mediated protein degradation. Proteins are targeted for degradation by the addition of a highly conserved poly-Ub chain, which is covalently attached to substrate proteins by a cascade system consisting of activating (E1), conjugating (E2), and/or ligating (E3) enzymes. An excision DNA repair Rad23 family proteins, including fission yeast Rhp23 [15] and human hHR23A/Rad23A, shuttle poly-Ub substrates to the proteasome for degradation [16]. Specifically, the Rad23 family proteins carry an ubiquitin-like (UbL) and two ubiquitin-associated (UbA) domains. A number of reports demonstrated that the UbA domains are important for binding of poly-Ub proteins whereas the UbL domain binds to proteasome [17]. However, it is currently unknown whether the role of hHR23A in promoting proteolysis is universal to all poly-Ub proteins or is specific to a subset of functionally relevant target proteins. During proteolysis, the 19S cap unfolds the Ub-tagged substrates and translocates them into the 20S catalytic core, where the proteins are degraded.

In this study, we took a unique approach in testing the interaction of Vpr with cellular proteins. Specifically, we started by using a fission yeast model system to search for genetic suppressors against nuclear import capacity of Vpr [18], [19]. Among other findings using this model system [20]–[22], one of the most intriguing discoveries was that Vpr interacts with the 19S proteasome through Rhp23/hHR23A. This was further confirmed by the fact that depletion of hHR23A interrupts interaction of Vpr with proteasome. Furthermore, Vpr promotes protein poly-ubiquitination *via* hHR23A. Most significantly, down-regulation of hHR23A reduces viral replication in non-dividing MAGI cells and macrophages, suggesting that hHR23A-mediated interaction of Vpr with proteasome plays an important role in viral replication in these non-dividing cells.

## Results

### Interaction of Vpr with the 19S regulatory subunit of the proteasome

#### Vpr can be displaced from the nuclear membrane in fission yeast by overexpression of cellular proteins that are associated with the 19S regulatory subunit of the proteasome

**Figure 1:**
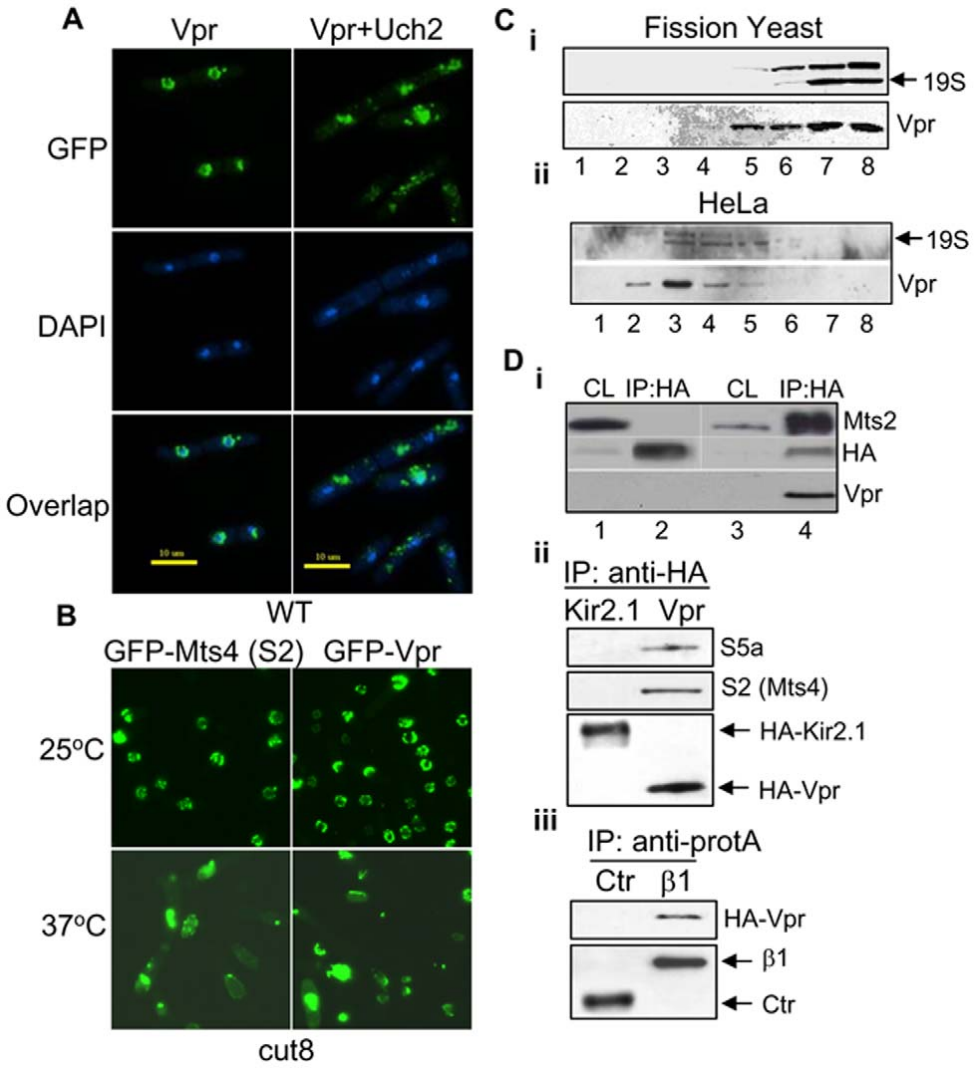
Vpr associates with proteasome in fission yeast and mammalian cells. **A**. Vpr is displaced from the nuclear membrane by overproduction of Uch2. Fission yeast cells carrying or not carrying Uch2 were stained with DAPI 17 hrs after *vpr* gene induction. Green color, GFP; Blue color, nuclear DNA. **B**. Vpr is displaced from the nuclear membrane in the *cut8* mutant. Mts4, a fission yeast homologue of mammalian S2, is a 19S proteasome-associated protein. Cut8 displays normal phenotype at the permissive 25°C, but shows mutant phenotype at non-permissive 37°C. **C**. Co-migration of Vpr with proteasome in fission yeast cells (**i**) and HeLa cells (**ii**) analyzed by glycerol gradient. Extracts from fission yeast cells expressing *vpr* were fractionated by centrifugation on a 10–40% glycerol gradient. Equal amounts of proteins from each fraction of the gradient were separated on 12% SDS-PAGE and probed with antibodies against Vpr and 19S (Mts4) subunits of the proteasome [34]. Lanes 1–8 indicates different fractions collected from the top (low molecular weight) to bottom of the gradient (high molecular weight). Note that not all fractions are shown here. **D.i**. Co-immunoprecipitation shows interaction of Vpr with Mts2 in yeast cells. IP was carried out with anti-HA as described previously [59]. A HA-tag alone plasmid control was used in this experiment. The recovered proteins were fractionated on SDS-PAGE and immunoblotted with anti-HA, anti-Vpr and anti-Mts2 antibodies. CL, cell lysates; IP:HA, immunoprecipitation with a HA-tagged control plasmid; IP:HA-Vpr, immunoprecipitation with a HA-Vpr carrying plasmid. **ii**. HeLa cells were transfected with HA-Vpr or HA-Kir2.1 (control). Kir2.1 is an irrelevant protein to Vpr and used here as a control. IP was carried out with anti-HA, recovered proteins were fractionated on SDS-PAGE and immunoblotted with anti-S2 (a mammalian homologue of fission yeast Mts4) and anti-S5a antibodies. **iii**. HeLa cells were co-transfected with pSG5-ZZ-β1, which codes for a proteasomal β1subunit [38], or control pSG5-ZZ plasmid (Ctr) together with HA-Vpr. The protein A-tagged β1 or control protein were pulled down by anti-protein A antibody, then blotted with anti-HA antibody.

Association of Vpr with nuclear envelope has been previously described by laboratories including ours both in fission yeast and mammalian cells [23]–[27]. The initial hint that Vpr interacts with proteasomes came from our search in fission yeast for multicopy suppressors of Vpr localization on nuclear membrane, an indication of the nuclear transport capacity of Vpr [28]. To search for such suppressors, a fission yeast cDNA library was expressed in the fission yeast RE078 strain containing a single copy of *gfp*-*vpr* gene integrated in the chromosome [29]. Approximately 2.0×10^4^ transformants, which statistically cover the entire yeast genome, were individually screened, and 21 unique clones were found to interfere with the nuclear localization of Vpr. Significantly, 8 of those clones encode proteins that associate with the 19S subunit of proteasome either directly (Rad25, Hsp70, Moc2, Pad1, Wos2 and Skp1) or indirectly (Uch2 and Phlp1) [30], [31]. As shown in **Fig. 1A**, cells expressing GFP-Vpr had intense green fluorescence at the nuclear rim with little labeling of the cytoplasm (**Fig. 1A**, top left). This pattern of nuclear membrane localization of Vpr observed in fission yeast cells has been reported previously, which is very similar to that observed in mammalian cells [20], [27], [32]. However, overexpression of Uch2 redistributed Vpr to the cytoplasm (**Fig. 1A**, top right). Similar displacement effects were also observed with other 19S-associated proteins (data not shown). These results indicate that overexpression of 19S proteasome-associated proteins affect Vpr localization, suggesting a possible association between proteasomes and Vpr.

#### Vpr detaches from the nuclear envelope when the proteasomes move away from the nuclear membrane

It has been well established that fission yeast proteasomes localize predominantly to the nuclear envelope during mitosis in wild-type yeast, but move away from the nuclear envelope to the cytoplasm in a *cut8* mutant strain [33], [34]. If Vpr associates with proteasomes on the nuclear membrane, it should also move away from the nuclear rim to the cytoplasm in the *cut8* mutant. To test this assumption, the localization of proteasomes (by using Mts4 as an indicator) and Vpr was monitored in live cells of the *cut8* mutant expressing the GFP-Vpr and GFP-Mts4 fusions. Mts4 encodes the subunit 2 (S2) of the 19S regulatory complex of the 26S proteasome [35] and it has been previously shown that Mts4 falls off the nuclear envelope along with the proteasome in the non-permissive temperature in the *cut8* mutant [33], [36]. As shown in **Fig. 1B**, both Vpr and Mts4 localized predominantly on the nuclear membrane at the permissive temperature of 25°C, when *cut8* cells behave like wild type cells. In contrast, at the non-permissive temperature of 37°C, Mts4 falls off the nuclear membrane as expected [**Fig. 1B**, bottom left; [33], [36]]. Similar to Mts4, predominant localization of Vpr to the nuclear rim is lost at the non-permissive temperature (**Fig. 1B**, bottom right), suggesting that nuclear membrane localization of Vpr correlates with proteasomes on the nuclear membrane.

#### The proteasome and Vpr co-migrate during centrifugation on a glycerol gradient in fission yeast and mammalian cells

To further test Vpr association with proteasomes, immunoblot analysis of fractions collected after centrifugation of cellular extracts of fission yeast (**Fig. 1C** **i**) and HeLa (**Fig. 1C** **ii**) cells on a glycerol gradient was performed. Fractionation on such gradient results in localization of the proteasome subunits in characteristic fractions [34]. Vpr, a small 15 kDa protein, is supposed to be at the top of the gradient in its free form, but much of the Vpr consistently migrated into the gradient and co-migrated with the 19S subunit fractions and indicated by anti-Mts4 (**Fig. 1Ci** and **1Cii**).

#### Confirmation of Vpr-proteasome interaction by co-precipitation

To test for a directly interaction between Vpr and proteasomes, a plasmid pSF173 carrying a HA-Vpr fusion was transfected into a wild type fission yeast strain and co-immunoprecipitation analysis was carried out with anti-HA antibodies. The precipitate was tested for Mts2, which is another subunit of the regulatory 19S complex of the 26S proteasome [35]. As shown in **Fig. 1D** **i**, Mts2 was detected in the pull down from the HA-Vpr-transfected strain (lane 4), whereas no Mts2 was pulled down in the HA-tag only control strain (lane 2), providing direct evidence for a physical interaction between Vpr and the proteasome.

To further confirm the interaction of Vpr and proteasomes in mammalian cells, the plasmids pcDNA3-HA-Vpr and pcDNA3-HA-Kir2.1, which contains an irrelevant HA-Kir2.1 fusion protein as control, were transfected into HeLa cells. The co-IP of HA-tag proteins were carried out with anti-HA antibody 48 hrs p.t. The precipitates were analyzed using anti-S2 and anti-S5a antibodies, both of which are components of the 19S subunit of the 26S proteasome [37]. In particular, S5a binds to hHR23A, a protein that was shown previously to bind Vpr [37]. As shown in **Fig. 1D** **ii**, both S2 and S5a were pulled down from pcDNA3-HA-vpr expressing HeLa cells, whereas no S2 or S5a was detected in control cells expressing Kir2.1.

In a reciprocal experiment, a pSG5-β1-ZZ plasmid, which contains an A-ZZ-tagged β1 subunit of the proteasome [38], or a control plasmid was co-transfected with pcDNA3-HA-Vpr into HeLa cells. The A-ZZ-tagged proteins were precipitated with anti-protein A antibody 48 hrs p.t. The pull-down cellular products were analyzed for Vpr by using anti-HA antibody. As shown in **Fig. 1D** **iii**, HA-Vpr was pulled down only in the A-ZZ-β1-expressing cells. Taken together, the above data provide strong evidence for specific interaction of Vpr with the 19S subunit of the proteasome in fission yeast and mammalian cells.

### Vpr binds to 19S proteasome *via* hHR23A

#### Interaction of Vpr with a fission yeast homologue Rhp23 of hHR23A

**Figure 2:**
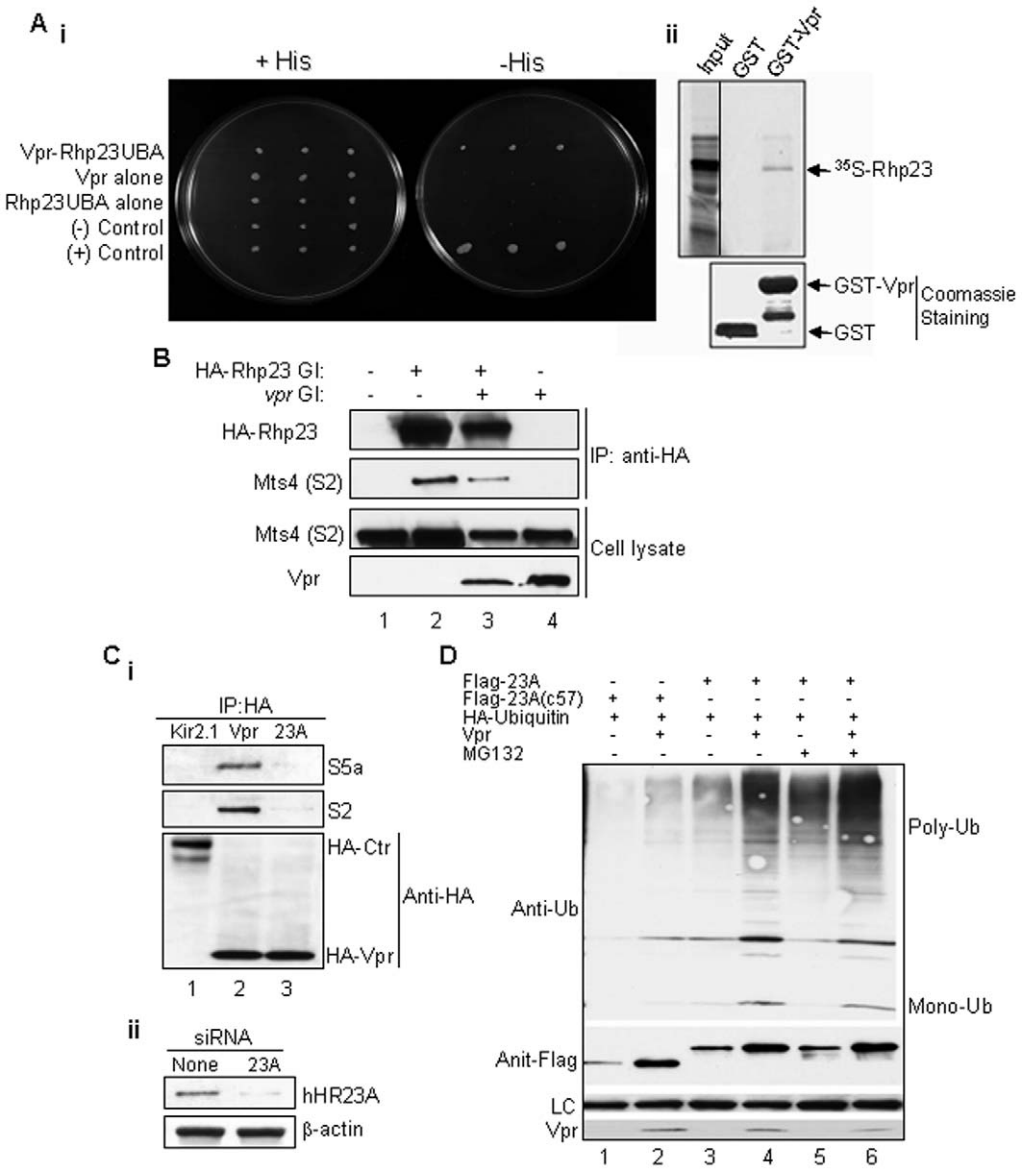
hHR23A is critical for Vpr-proteasome interaction. **A**. *In vitro* and *in vivo* interactions of Rhp23 with HIV-1 Vpr. (**i**) Rhp23, a fission yeast homologue of mammalian hHR23A[15], interacts with Vpr in the yeast two-hybrid system. The *vpr* gene was inserted into the pGBT9 plasmid and *rph23* was fused to the activation domain in the pGAD-GH plasmid. The interaction was measured by β-galactosidase assay on cell extracts. (**ii**) *In vitro* interaction of Vpr with Rhp23. Bacterial cell lysates over-expressing GST and GST-Vpr proteins were immobilized on GST-agarose beads. *In vitro* translated 35S-labeled Rhp23 (shown by arrow) was incubated with the immobilized GST and GST-Vpr. The Coomassie staining (low panel) shows total proteins, and autoradiography (top panel) shows bound 35S-labeled Rhp23. **B**. Interaction of Rph23 with proteasome in the presence or absence of Vpr. HA-Rhp23-carrying plasmid was transfected into fission yeast in the presence or absence of Vpr. Following immunoprecipitation with anti-HA antibody, precipitates were tested with anti-Mts4, which recognizes the 19S regulatory subunit of the proteasome. **C**.**i**. Depletion of hHR23A abolished the interaction of Vpr with proteasome in HeLa cells. The HA-Vpr or HA-Kir2.1 (control) expressing vectors were transfected into HeLa cells with (lane marked 23A) or without hHR23A depletion by siRNA. The HA-tagged proteins were pulled down by anti-HA antibody, and then blotted with anti-S2 and anti-S5a antibodies, which recognize S2 and S5a, respectively, of the 19S regulatory subunits of the proteasome. **ii**. 50 µg of supernatants from lanes 2 (None) and 3 (23A) of **a** were blotted with anti-Rad23A or β-actin antibody. **D**. Vpr promotes protein poly-ubiquitination *via* hHR23A. Flag-tagged hHR23A was co-expressed with HA-ubiquitin in the presence or absence of Vpr in HeLa cells. Forty-eight hours after transfection, cells were collected and cell extracts were subject to Western blot analysis using indicated antibodies. hHR23(c57) is a non-functional mutant derivative of hHR23A [43]. MG132 was used to inhibit the proteasome activities.

Earlier studies demonstrated a physical interaction between Vpr and hHR23A, a human protein that contains a N-terminal UbL and 2 C-terminal UbA domains [37], [39]. Further analysis of this interaction indicated that Vpr binds to hHR23A through its C-terminal UbA domain [40]. Previous studies on this protein and its budding yeast orthologue Rad23 showed that hHR23A homologues bind to the proteasome via the N-terminal UbL domain [41], [42]. However, the biological function(s) of the Vpr-hHR23A interaction and the significance of UbA or UbL interactions with Vpr or proteasome during the Vpr-hHR23A were unknown. To address these questions, we cloned and characterized a fission yeast orthologue (Rhp23) of human hHR23A [15]. Same as hHR23A, Rhp23 also contains both the N-UbL and the two C-UbA domains. Consistent with the Vpr-hHR23A interaction, using the yeast two-hybrid system we demonstrated that Rhp23 also binds to Vpr through the UbA domain [**Fig. 2A** **i**]. Furthermore, *in vitro* interaction of Vpr with Rhp23 was further verified by incubating the bacterial cell lysates over-expressing GST or GST-Vpr protein with 35S-labeled Rhp23∶Rhp23 bound only to GST-Vpr protein (**Fig. 2A** **ii).**

#### Same as hHR23A, Rhp23 also associates with proteasome

To confirm the association of Rhp23 with proteasome in fission yeast, a plasmid containing HA-tagged Rph23 was transfected into fission yeast with or without *vpr* expression. Following immunoprecipitation with anti-HA antibody, anti-Mts4, which recognizes the S2 subunit of the 19S regulatory complex of the proteasome, was used to detect proteasome. As shown in **Fig. 2B**, Mts4 protein was detected in the HA-Rph23 pulled down protein complexes regardless of whether Vpr was absent or present (**Fig. 2B**, lanes 2,3).

#### Depletion of hHR23A by siRNA reduces binding of Vpr to proteasome

Since Vpr appeared to interact with proteasome through Rhp23 or hHR23A in fission yeast and mammalian cells, we were interested in determining whether hHR23A is indeed required for Vpr interaction with 26S proteasome. The same experiment as shown in **Fig. 1D** **ii** was repeated, except that hHR23A was also depleted in one of the samples using siRNA. Specifically, the pcDNA3-HA-Vpr was transfected into HeLa cells with or without hHR23A mRNA knockdown by specific siRNA. Co-IP of HA-tagged proteins was conducted using anti-HA antibody and presence of proteasome in the precipitates was detected using anti-S2 and anti-S5a antibodies. As expected, both S2 and S5a were detected in the pull-down protein extracts of vpr-expressing HeLa cells (**Fig. 2C** **i**, lane 2). In contrast, little or no S5a and S2 were seen in the hHR23A-depleted cells (**Fig. 2C** **i**, lane 3). Depletion of hHR23A was confirmed by Western blot analysis (**Fig. 2C** **ii**). Together, these data suggest that Vpr binds to 19S complex of proteasome at least in part through hHR23A.

#### Vpr promotes protein poly-ubiquitination *via* hHR23A

Since Vpr interacts with proteasome through hHR23A, we were interested in learning the potential effect of Vpr on the role of hHR23A in protein poly-ubiquitination. To address this question, the Flag-tagged hHR23A was co-expressed with HA-ubiquitin in the presence or absence of Vpr in HeLa cells. Forty-eight hours after transfection, cells were collected and cell extracts were subjected to Western blot analysis using anti-HA antibody. The Flag-tagged hHR23A(c57), a mutant derivative of hHR23A that contains only the COOH-terminal 57 amino acid residues [43], was used as a negative control. Same as in fission yeast (data not shown), expression of hHR23A significantly increased the amount of poly-Ub in comparison with cells expressing the hHR23A(c57) mutant (compare lane 3 to lane 1 in **Fig. 2D**). This hHR23A-mediated poly-Ub was further enhanced by a proteasome inhibitor, MG132 (compare lanes 3 and 5 in **Fig. 2D**). Thus the expression of Vpr appeared to promote hHR23A-mediated protein poly-Ub (**Fig. 2D**, lanes 2, 4 and 6). The low level of ubiquitination in cells transfected with hHR23A(c57) mutant was likely mediated by endogenous hHR23A. Together, these data support the notion that Vpr promotes protein poly-Ub *via* hHR23A.

#### Depletion of hHR23A significantly reduced HIV-1 replication in a Vpr-dependent fashion

**Figure 3:**
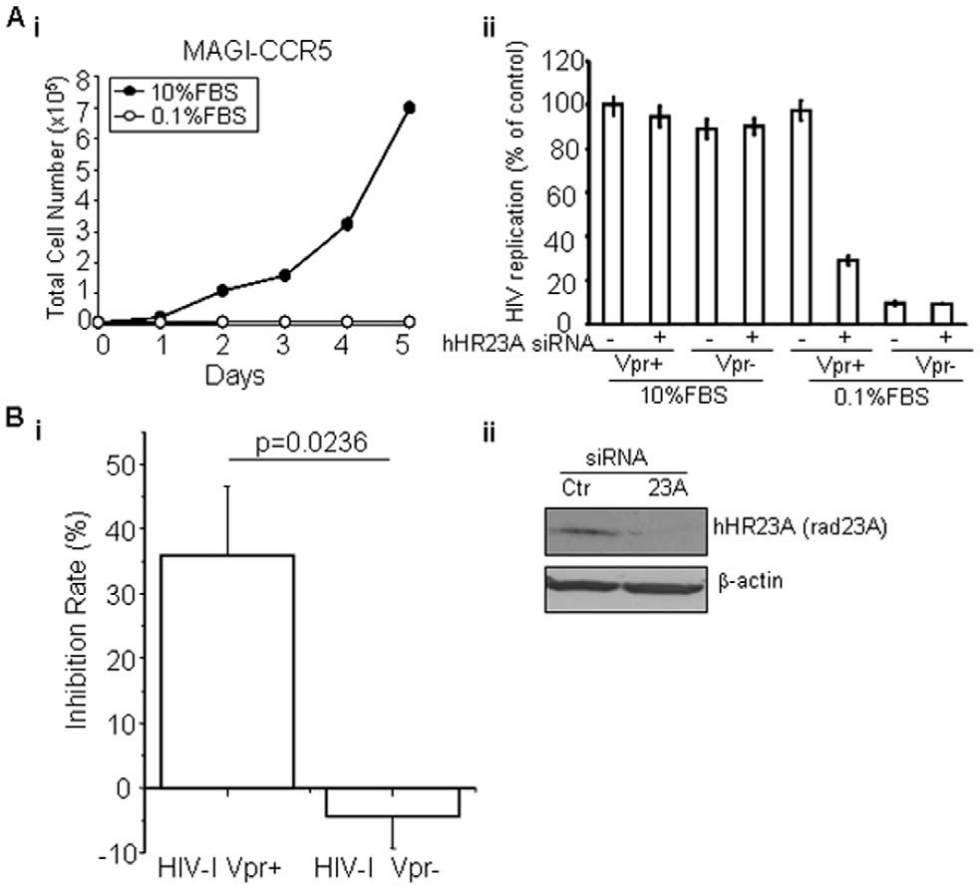
hHR23A is required for Vpr-mediated stimulation of HIV-1 replication in non-dividing MAGI-CCR5 cells and macrophages. **A.i.** Cell proliferation of MAGI-CCR5 cells in different FBS concentration. MAGI-CCR5 cells were plated at 7,000 cells/well in DMEM containing 10% or 0.1% of FBS. Cellular proliferation was measured over a period of 5 days. Cells in 0.1% FBS were viable over the experimental period, as they remained adherent to plates. **ii.** Depletion of hHR23A significantly reduces viral replication in non-dividing MAGI cells in a Vpr-dependent manner. MAGI-CCR5 cells were transfected with hHR23A-targeting (+) or control (−) siRNA, plated in DMEM with 10% or 0.1% FBS, and infected with HIV-1_Ada_ Vpr(+) or Vpr(−) 24 hrs after hHR23A knockdown. Viral replication was evaluated 48 hrs after infection by staining; blue cells were counted as infected. Results are presented as percent of control, i.e., the number of blue cells in cultures transfected control siRNA and infected with Vpr-positive HIV-1 and show average ± SE of quadruplicate determinations. **B.** Vpr-dependent HIV-1 viral replication in macrophages is mediated through hHR23A. **i.** Monocyte-derived macrophages pretreated with hHR23A siRNA or control (Ctr) siRNA were infected with HIV-1_Ada_ (Vpr+) or (Vpr−) viruses. Cells were collected 72 hrs *p.i.* and viral replication was determined by measuring p24. Results are presented as inhibition of HIV-1 replication in cells treated with hHR23A siRNA relative to cells treated with control siRNA, and show mean ± SE of three independent experiments with cells from different donors, each performed in triplicate. Statistical analysis was performed using Student's t-test, and *p* value is shown. **ii.** Monocyte-derived macrophages were transfected with hHR23A siRNA or control siRNA. Cells were collected 72 hrs *p.t.* and subjected to Western blot analysis using anti-Rad23A and anti-β-actin antibodies.

Since HIV replication-stimulating activity of Vpr is most evident in non-dividing cells [1], [44], we decided to first compare the potential effect of hHR23A depletion on Vpr-dependent viral replication in proliferating and non-dividing cells. hHR23A was knocked down in MAGI-CCR5 cells using siRNA (control cells were treated with control siRNA), cells were transferred from high (10% FBS) to low serum (0.1% FBS) to stop cell proliferation [45] and both proliferating and non-dividing cells were then infected by Vpr-positive (Vpr+) or Vpr-negative (Vpr−) HIV-1_Ada_. The cell proliferation status in high or low serum was examined by measuring growth curves. As shown in **Fig. 3A** **i**, MAGI cells cultured in normal high serum proliferated exponentially as expected; in contrast, no growth was detected over a period of 5 days after MAGI cells were transferred to 0.1% FBS. Even though serum-starved cells showed no cellular proliferation, no obvious cell death was observed as all the cells remained adherent to the bottom of the culture plates (data not shown).

For HIV-1 infection, both control and hHR23A-depleted cells were infected with HIV-1_Ada_ Vpr(+) or Vpr(−) virus with TCID_50_ of 3,000 24 hrs after the hHR23A knockdown. Viral replication was evaluated 48 hrs after infection by staining for X-Gal expression; blue cells were counted as infected cells. To avoid potential biases in counting HIV-infected blue cells, 4 independent experiments were carried out and each experiment was counted independently by two individuals. As shown in **Fig. 3A** **ii**, there are no significant differences in the levels of viral replication between wild type and hHR23A-depleted cells in normally proliferating cells; as expected, a small but appreciable decrease of viral replication was seen in cells infected with Vpr(−) virus. In the non-proliferating cells, however, there were profound differences in the level of viral replication between Vpr(+) and Vpr(−) viruses. Replication of the Vpr-negative virus was about 90% lower than of the Vpr(+) HIV-1. Most significantly (*p*<0.05), depletion of hHR23A reduced replication of the Vpr(+) virus by over 65% (**Fig. 3A** **ii**), whereas no effect was observed on replication of the Vpr(−) HIV-1.

We next performed a similar experiment with monocyte-derived macrophages. The isolated macrophages were transfected with hHR23A siRNA or control siRNA, infected with HIV-1_Ada_ (Vpr+) and (Vpr−) viruses, and transfected again. Cells were collected 72 hrs *p.i.* and HIV-1 replication was determined by measuring p24. The observed reduction of viral replication due to hHR23A was depicted as the % of inhibition rate. As shown in **Fig. 3B** **i**, while no obvious effect of hHR23A suppression was found in macrophages infected with the Vpr(−) viruses, hHR23A-depleted cells showed an approximately 40% reduction of viral replication (*p*<0.05). Successful downregulation of hHR23A mRNA was demonstrated by Western blotting using anti-Rad23A antibody (**Fig. 3B** **ii**). Note that statistically significant differences were found in the hHR23A knock-down cells in MAGI-CCR5 (**Fig. 3A** **ii**) and macrophages (**Fig. 3B**). Time-course experiments with multiple MOIs were not conducted. Thus contribution of hHR23A under other experimental conditions is unknown.

Together, results of these experiments strongly suggest that the stimulating effect of Vpr on HIV-1 replication in non-dividing cells is due, at least in part, to hHR23A-mediated Vpr-proteasome interaction.

## Discussion

In this study, we have demonstrated for the first time that Vpr interacts directly with the 26S proteasome. We have further shown that this interaction is linked to the regulatory 19S subunit of the proteasome through hHR23A, a Vpr-binding protein that is capable of shuttling ubiquitinated proteins to the proteasome for degradation [16]. Our results further show that Vpr promotes protein-ubiquitination, thus it may potentially lead to enhanced protein degradation of the downstream targets. While those downstream targets are currently unknown, it is clear that this hHR23A-mediated interaction of Vpr with proteasome plays a significant role in HIV-1 replication in non-proliferating MAGI-CCR5 cells and primary macrophages.

The major function of the 26S proteasome is to digest proteins that are tagged for degradation. During host-pathogen interactions, the proteasome-mediated proteolysis is often used both by host cells to restrict viral infection and/or by the pathogen to counteract host restriction. For example, proteasome-mediated proteolysis is involved in the host cell MHC class I antigen presentation for viral antigen processing. The viral proteins are broken down by the proteasome so that the viral epitopes can be recognized by CD8 T-lymphocytes to trigger cytotoxic T-lymphocyte (CTL) response, a process that specifically destroys the infected cells. Conversely, proteasome-mediated proteolysis is also required for viral survival and pathogenesis. HIV-1 Tat protein binds to the 26S proteasome and inhibits its activity [46]. Similarly, SIV_SM_/HIV-1 Vpx proteins promote retroviral escape from a proteasome-dependent restriction pathway present in human dendritic cells [47].

Several HIV-1 proteins are known to be involved in protein degradation through mediation of protein ubiquitination *via* binding to Ub E3 ligases. For example, HIV-1 Vif is known to promote degradation of the host cellular antiviral factor, APOBEC3G, through interaction with a Cullin5-ElonginBC Ub E3 ligase complex [48], [49]. Similarly, Vpr binds to a specific Cullin Cul4A-DDB1-DCAF1/VprBP ubiquitin E3 ligase for induction of cell cycle G2 arrest in proliferating cells [50], [51]. Thus, it is possible that Vpr may facilitate degradation of a specific subset of target proteins by first engaging the VprBP-associated E3 ligase for protein tagging with Ub; the ultimate protein degradation might be mediated by Vpr, for example, through shuttling of ubiquitinated proteins to proteasome *via* hHR23A.

Binding of Vpr to hHR23A has been reported previously [37]. Earlier studies on the interaction of Vpr with hHR23A suggested that this interaction might play a role in the induction of G2 arrest [37], [39], but mutational analysis did not support this interaction [52]. Thus, the functional role of Vpr-hHR23A interaction in HIV-1 infection has not been resolved. Results presented in this report suggest that the Vpr-hHR23A interaction might be involved in proteasome-mediated proteolysis. Interestingly, while we were preparing for this report, a new report came out [53] and showed that Vpr induces protein polyubiquitination of unknown proteins through direct protein-protein interactions. Furthermore, these interactions appear to correlate with Vpr-induced cell cycle G2 arrest and the proteasome activity. G2-arrest-defective mutants of Vpr decreased those interactions; whereas inhibition of proteasomal activity enhanced them. Thus, it would be of great interest to test whether hHR23A is among those polyubiquitinated proteins and further to re-visit its role in Vpr-induced G2 arrest. It should be mentioned that this recent report [53] did not show direct interaction of Vpr with proteasome and the specific involvement of hHR23A in this interaction. Thus, we believe that interaction of Vpr with hHR23A may underlie a new biological activity of Vpr in which Vpr associates with and affects the activity of the proteasome.

One of the most intriguing findings of this study is the contribution of hHR23A-Vpr interaction to viral replication in non-dividing cells but not in proliferating cells (**Fig. 3**). Even though requirement of Vpr in HIV-1 infection of non-dividing cells is not new [8], the molecular action of Vpr in this process is not well understood. It has been long believed that the reason why Vpr is required for viral replication in cells such as macrophages is that Vpr promotes nuclear import of PIC in non-dividing cells [1]. However, a recent study demonstrated that the differential requirement of Vpr may not be due to the cell proliferation status [9]. In addition, Vpr also participates in nuclear import of PIC in T cells in a similar manner as it does in macrophages, and nuclear import through the nuclear pore is essential for HIV replication in both cell types [10]. An alternative possibility is that cellular factors that either regulate Vpr or are regulated by Vpr may differ between proliferating and non-proliferating cells and thus determine whether Vpr is needed. For example, expression of a cellular factor HSP70, which can substitute for the nuclear import activity of Vpr, is higher in T-cells than in macrophages, thus diminishing dependence of viral replication on Vpr in T-cells [54].

Several recent reports demonstrate that the activity of Vpx, also limited to macrophages, stimulates reverse transcription by counteracting a yet unidentified cellular restriction factor [11], [55]. Vpx is absolutely essential for SIV replication in macrophages, and expression of Vpx stimulates replication in these cells in the context of a number of retroviruses, including HIV-1 and MLV [13]. The finding that Vpx stimulates replication in macrophages of Vpr-expressing HIV-1 [11] suggests that either Vpr is a weaker inhibitor of a Vpx-targeted restriction factor, or that Vpr's target is different from that of Vpx. Interestingly, HIV-2 Vpx binds to the same VprBP-associated E3 ligase as HIV-1 Vpr to overcome restriction factors in macrophages [11], [12], [55]. The ability of Vpx to counteract the restriction of HIV-1 and SIV infection depends on DDB1, a subunit of the VprBP-associated E3 ligase. A DDB1-Vpr fusion could partially substitute for the role of Vpx [11]. These findings are consistent with our model that Vpr may work in concert with an ubiquitin-proteasome system to limit cellular factor(s) restricting HIV replication in non-dividing cells: interaction with such factors as DDB1 (for Vpx) or hHR23A (for Vpr) may define the target and fine-tune the viral protein activity.

**Figure 4:**
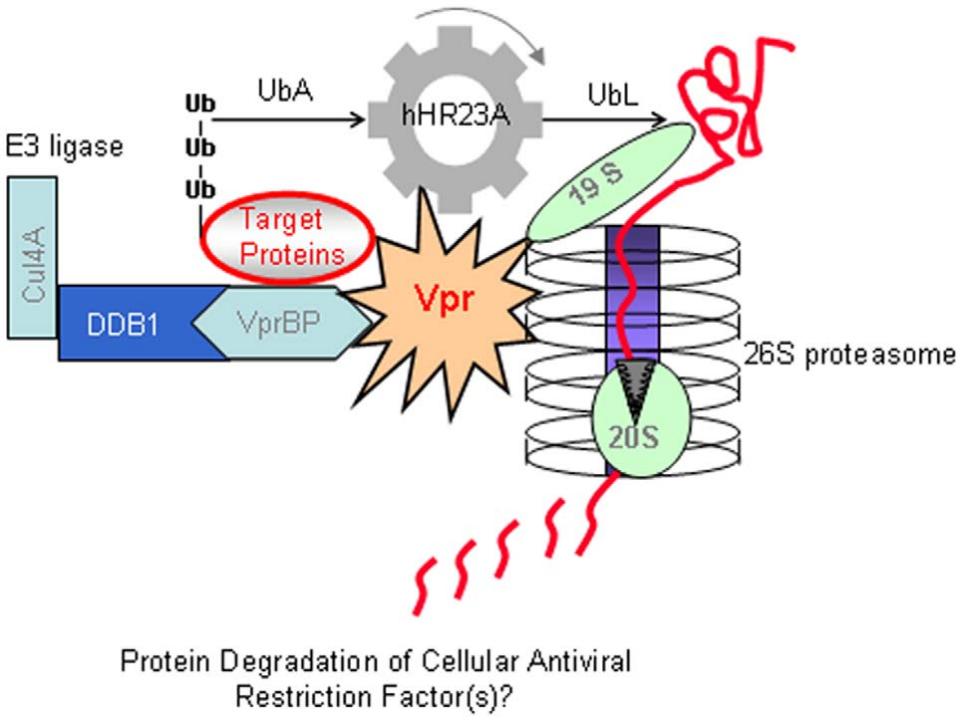
Proposed model of Vpr-proteasome interactions. Details are in the text.

Thus, we propose that Vpr plays a role similar to that of Vpx, i.e., it promotes inactivation of a restriction factor expressed in macrophages and some non-dividing cells through an ubiquitin-proteasome process. **Fig. 4** depicts a working model to describe our current understanding of the role Vpr-hHR23A interaction plays in Vpr-mediated stimulation of HIV-1 replication in macrophages. First, cellular proteins may be targeted by Vpr *via* binding of these proteins to the Vpr-VprBP-associated E3 ligase complex, in which VprBP is an adaptor protein for the E3 and is responsible for the substrate specificity. Second, Vpr interaction with hHR23A fine-tunes the binding of hHR23A UbA domains to poly-Ub proteins, which are subsequently shuttled to the 26S proteasome *via* binding of hHR23A UbL domain to 19S subunit. Upon receiving the Ub-tagged proteins, the 19S cap of the proteasome unfolds the Ub-tagged target proteins and translocates them into the 20S catalytic core, where the proteins are ultimately degraded. We propose that the repertoire of proteins targeted by Vpr through this mechanism differs between different cell types, and specifically between T cells and macrophages, thus explaining a more potent effect of Vpr in the latter cells. Results generated from testing this working model will provide additional insights into the specific role of Vpr in viral replication.

## Materials and Methods

### Yeast strains and mammalian cells

All of the Fission yeast (*Schizosaccharomyces pombe*) strains, human cell lines, plasmids and HIV-1 viral stocks used in this study are shown in **Table 1**. Yeast cells were grown in complete medium containing adenine (YEA) or Edinburgh minimal medium (EMM) by using standard culture techniques [56]. Human HeLa cells and MAGI-CCR5 cells were maintained in Dulbecco's modified Eagle's medium (DMEM) (Cellgro) supplemented with 10% fetal bovine serum (FBS, Invitrogen). Human monocyte-derived macrophages were prepared from peripheral blood mononuclear cells by plastic adherence and cultivated using standard techniques as described previously [44].

**Table 1:** Fission yeast strains, human cell lines, plasmids and HIV-1 viral stocks.

Strains/Plasmids	Genotype and Characters	Source or Reference
S. pombe strains:		
SP223	Wild type, *h^−^, ade6-216, leu1-32, ura4-294*	[26]
RE078	SP223 with single copy of *gfp*-*vpr* integrated at *ura4* locus; used for determining nuclear localization of Vpr	[20]
Cut8	*h-*, *leu1*, *ura4*, Δ*cut8*::*ura4*+	[33]
Mammalian cell lines:		
HeLa	A cervical epithelial cell line	ATCC
MAGI-CCR5	A CD4-positive derivative of HeLa cell line; containing an integrated HIV-1 LTR-driven β-galactosidase reporter gene	NIH AIDS Research and Reference Reagents Program
S. pombe Plasmids:		
pYZ1N	Fission yeast expression vector with an inducible *nmt1* promoter and a *leu1* selectable marker	[26]
pYZ3N	Same as pYZ1N but with a 5′ GFP-tag	[26]
pSF173	Fission yeast expression vector with an inducible *nmt1* promoter and a 5′ HA-tag, *ura4* selectable	ATCC
Mammalian plasmids:		
pCDNA3.1	Mammalian expression vector with a CMV promoter; hygromycin-resistant	Invitrogen
pcDNA3.1-HA-Vpr	HA-tagged Vpr on pcDNA3.1	Nathaniel R. Landau
pCDNA3-HA-Kir2.1	HA-tagged Kir2.1., a control plasmid for HA-Vpr	Paul Weilling
pSG5-β1-ZZ	Proteasomal β1 subunit cloned in the pSG5-ZZ plasmid	[38]
pSG5-ZZ	A control plasmid for pSG5-β-ZZ	[38]
HIV-1 viral stocks:		
HIV-1_Ada_ Vpr (+)	Packaged virus using the HIV-1 Ada strain with wild type Vpr	[20]
HIV-1_Ada_ Vpr (−)	Packaged virus using the HIV-1 Ada strain with a mutant Vpr	[20]

### Gene expression in fission yeast cells

Gene induction under the control of the fission yeast *nmt1* promoter in liquid medium has been described previously [56]. Briefly, cells containing the plasmid with the *nmt1* (**n**o **m**essage in **t**hiamine) promoter were first grown to stationary phase in the presence of 20 µM thiamine. Cells were then washed three times with distilled water, diluted to a final concentration of approximately 2×10^5^ cells/ml in 10 ml of the appropriately supplemented EMM medium with or without thiamine. Cells were examined 24 hours after gene induction. All cells were normally grown at 30°C with constant shaking at 200 rpm unless otherwise specified.

### Transfection of mammalian cells with plasmids and siRNA

All of the plasmids were transfected into cells by using Lipofectamine 2000 following manufacture's instructions (Invitrogen). HPLC-purified siRNAs commercially designed to specifically target hHR23A (Cat. No. SI02663654), and control non-silencing siRNA (Cat. No. 1022083) were purchased from Qiagen (Valencia, CA). The siRNA mixture was transfected at a concentration of 10 nM into approximately 5×10^5^ HeLa cells by use of 8 µl of Lipofectamine RNAiMAX following manufacturer's instructions (Invitrogen). Measurement of transfection efficiency by using Rhodamine labeled siRNA indicated an efficiency of over 90% (data not shown).

For transfection of primary monocyte-derived macrophages, 1×10^6^ cells were incubated with 150 pmol of siRNA and 7 µl metafectene (Biontex) in Opti-MEM (Gibco) at 37°C for 3–5 hours. On day 2, cells were infected with HIV-1_Ada_, and on day 3 transfection procedure was repeated.

### Fluorescence microscopy

A Leica DMR fluorescence microscope (DM4500B; Leica Microsystems) equipped with a high-resolution camera (Hamamatsu) and OpenLab software (Improvision) was used for all of the imaging analysis. For analyzing the subcellular localization of green fluorescent protein (GFP)-tagged fusion proteins in fission yeast, live cells were observed under a fluorescence microscope and images were captured 18 to 20 h after *vpr* gene induction. Nuclear localization was verified by DNA staining with 1 µg of DAPI (4′, 6′-diamidino-2-phenylindole)/ml.

### Yeast 2-hybrid system

The vpr gene was inserted into the pGBT9 plasmid and rph23 was fused to the activation domain in the pGAD-GH plasmid. The strength of the interaction was measured by β-galactosidase assays on cell extracts as described previously [57].

### *In vitro* interaction of Vpr with Rhp23

Bacterial cell lysates over-expressing GST and GST-Vpr proteins were immobilized on GST-agarose beads. In vitro translated 35S-labeled Rhp23 was incubated with the immobilized GST and GST-Vpr. After extensive washes, the bound proteins were eluted and separated by SDS-PAGE, then subjected to coomassie staining or autoradiography detection.

### Cell lysis and immunoblotting analysis

For fission yeast, cell lysates were prepared by the glass beads method using lysis buffer (50 mM Tris-HCl, pH 8.0; 50 mM NaF; 1 mM Na3VO4; 5 mM EDTA; 150 mM NaCl; 10% glycerol; 0.1% Triton X-100) supplemented with protease inhibitors as previously described. Mammalian cells were lysed with lysis buffer (50 mM Tris, pH 7.5; 150 mM NaCl; 2 mM EDTA; 1% Triton X-100) on ice for 30 min and the debris was removed by centrifugation at 13,000 rpm for 1 min. The protein concentrations of supernatants were measured by BCA protein assay kit (Pierce).

For Western blot analysis, 30 to 50 µg of protein was loaded on 10–20% gradient Criterion Precast Gels (BioRad) for electrophoretic separation. Proteins were transferred to the Trans-blot® Nitrocellulose membranes and blotted with 5% skim milk in TBST buffer (10 mM Tris, pH 8.0; 150 mM NaCl; 0.1% Tween 20) for 30 min at room temperature. Primary antibodies were then applied overnight at 4°C. After washing 3 times in TBST for 10 min each time, the membranes were incubated with secondary antibody for 1 h at room temperature. Membranes were washed again and proteins were detected with Supersignal® Western Dura Extended Duration Substrate (Pierce, Rockford, IL)[47]. The following primary antibodies were used: mouse monoclonal anti-hemagglutinin antibody (anti-HA; HA-7, Sigma), mouse monoclonal anti-Flag antibody (M2, Sigma), mouse monoclonal anti-β-actin antibody (AC-15, Sigma), rabbit polyclonal anti-Protein A antibody (Sigma), rabbit polyclonal anti-19S proteasome S2 subunit antibody (Calbiochem), rabbit polyclonal anti-19S proteasome S5a antibody (Calbiochem), rabbit anti-Rad23A antibody (H-87, Santa Cruz Biotechnology); rabbit polyclonal anti-Ubiquitin antibody (Cell Signaling); rabbit polyclonal anti-Vpr serum was custom generated by the Proteintech Group, Inc. (Chicago, IL). Goat anti-mouse horseradish peroxidase-conjugated and goat anti-rabbit horseradish peroxidase-conjugated antibodies were used as secondary antibodies (Bio-Rad). Antisera against fission yeast Mts4 and Mts2 were gift from Dr. Colin Gordon as described previously [35].

### Protein co-migration analysis

Co-migration of Vpr with proteasome was analyzed by glycerol gradient in fission yeast and HeLa cells. Extracts from fission yeast and HeLa cells expressing vpr were fractionated by centrifugation on a 10–40% glycerol gradient. Equal amounts of proteins from each fraction of the gradient were separated on 12% SDS-PAGE and detected with antibodies against Vpr and 19S subunits of the proteasome by using anti-Mts4 antibody [58].

### Co-immunoprecipitation

The co-immunoprecipitation of HA-tag proteins for fission yeast were carried out as previously described [59]. Briefly, proteins were immunoprecipitated from the cell lysates with anti-HA antibody overnight at 4°C. The protein-antibody complexes were subsequently collected by adding protein A-agarose beads and incubated for 2 hrs at 4°C. Immunoprecipitates were then washed three times with PBS containing protease inhibitors prior to analysis.

For mammalian cells, the co-immunoprecipitation of HA-tag proteins was carried out with ProFound Mammalian HA Tag IP/Co-IP kit according to manufacturer's instructions (Cat.No. 23615, PIERCE). Briefly, cells were lysed with M-PER mammalian protein extraction reagent on ice for 30 min and the debris was removed by centrifugation at 13,000 rpm for 1 min. The cell lysate was transferred to spin column and 6 µl anti-HA agarose slurry was added into each cell lysate. After incubation with gentle end-over-end mixing overnight, supernatant was collected into collection tube with pulse centrifugation for 10 seconds. The column containing HA-tagged proteins was washed three times with 0.5 ml TBST, then eluted with 60 µl of 2X non-reducing sample buffer at 95–100°C on a heat block for 5 minutes. The eluted proteins were collected by pulse centrifugation for 10 seconds, and 3 µl of 2-mercaptoethanol was added for SDS-PAGE.

### MAGI assay

MAGI assay was used to determine the viral infectivity. Briefly, MAGI-CCR5 cells were seeded in 6-well plates at 7,000 cells per well. On the following day, cells were transfected with specific siRNA against hHR23A or control siRNA. Both control and hHR23A-depleted cells were infected with HIV-1_Ada_ Vpr(+) or Vpr(−) virus with TCID_50_ of 3,000 24 hrs after hHR23A knockdown. The media were removed and cells were fixed by 2 ml fixing solution (1% formaldehyde, 0.2% glutaraldehyde in PBS) 48 hrs after infection. Viral replication was evaluated by staining with 600 µl of staining solution (6.6 mM potassium ferrocyanide, 3.3 mM MgCl_2_, 0.7 mg/ml X-Gal in PBS). Blue cells were counted as infected cells in each well under microscope.

### Infection of macrophages

Monocyte-derived macrophages were inoculated with HIV-1_Ada_ (Vpr+) and (Vpr−) viruses at 4×10^6^ cpm of RT activity/10^6^ cells in 200 µl of medium, centrifuged for 1 h at 2,000 rpm at room temperature, and incubated for 3 hours at 37°C, followed by 3 washes with PBS. Infected cells were cultivated in fresh RPMI 1640 complete medium supplemented with 10% human serum. Every 3–4 days, half of the medium was changed and checked for p24 activity.
